# Changes in chemical coding of sympathetic chain ganglia (SChG) neurons supplying porcine urinary bladder after botulinum toxin (BTX) treatment

**DOI:** 10.1007/s00441-014-2086-3

**Published:** 2015-01-27

**Authors:** E. Lepiarczyk, A. Bossowska, M. Majewski

**Affiliations:** Department of Human Physiology, Faculty of Medical Sciences, University of Warmia and Mazury in Olsztyn, Warszawska 30, 10-082 Olsztyn, Poland

**Keywords:** Sympathetic chain ganglia (SChG), Botulinum toxin (BTX), Urinary bladder, Immunohistochemistry, Pig

## Abstract

Botulinum toxin (BTX) is a neurotoxin used in medicine as an effective drug in experimental therapy of neurogenic urinary bladder disorders. We have investigated the influence of BTX on the chemical coding of sympathetic chain ganglia (SChG) neurons supplying the porcine urinary bladder. The toxin was injected into the wall of the bladder. SChG neurons were visualized by a retrograde tracing method with fluorescent tracer fast blue (FB) and their chemical coding was investigated by double-labelling immunohistochemistry with antibodies against dopamine β-hydroxylase (DβH; a marker of noradrenergic neurons), neuropeptide Y (NPY), vasoactive intestinal polypeptide (VIP), somatostatin (SOM), galanin (GAL), Leu^5^-enkephalin (L-ENK) and neuronal nitric oxide synthase (nNOS). In both the control (*n* = 5) and BTX-treated pigs (*n* = 5), the vast majority (91 ± 2.3 % and 89.8 ± 2.5 %, respectively) of FB-positive (FB+) nerve cells were DβH+. BTX injections caused a decrease in the number of FB+/DβH+ neurons that were immunopositive to NPY (39.5 ± 4.5 % vs 74.5 ± 11.9 %), VIP (8.9 ± 5.3 % vs 22.3 ± 8.8 %), SOM (5.8 ± 2.3 % vs 17.4 ± 3.7 %) or GAL (0.9 ± 1.2 % vs 5.4 ± 4.4 %) and a distinct increase in the number of FB+/DβH+ neurons that were immunoreactive to L-ENK (3.7 ± 2.9 % vs 1.1 % ± 0.8 %) or nNOS (7.7 ± 3.5 % vs 0.8 ± 0.6 %). Our study provides novel evidence that the therapeutic effects of BTX on the mammalian urinary bladder are partly mediated by SChG neurons.

## Introduction

Botulinum toxin (BTX), produced by the anaerobic bacterium *Clostridium botulinum* (van Ermengem 1897), is one of the most potent neurotoxins known. It acts by entering the presynaptic bulb of cholinergic neurons and by inhibiting acetylcholine (ACh) release as a result of the prevention of normal vesicle docking and fusion to the presynaptic plasma membrane (Haferkamp et al. 2004). To date, the toxin has been proposed for the treatment of numerous neurogenic dysfunctions, characterized by excessive or inappropriate muscle contractions, such as strabismus, blepharospasm or muscular dystonias (for reviews, see Carruthers and Carruthers 2004; Lowe and Lowe 2012).

For several years, BTX has also been used as an effective drug in experimental therapies of a range of neurogenic urinary bladder disorders and, since the end of 2011, it has been approved for the treatment of urinary incontinence secondary to neurogenic detrusor activity (Cruz et al. 2011). The literature in the field contains many contributions regarding the possible clinical use of BTX in urology (see Rohrsted et al. 2012; Pinto et al. 2013; Santos-Silva et al. 2013).

Because of the mechanism of its action, BTX apparently influences parasympathetic/cholinergic neurons that supply the urinary bladder and that, through the release of ACh, activate muscarinic receptors and mediate the contraction of the detrusor muscle (Thüroff et al. 1982), thereby causing bladder emptying and micturition (Levin et al. 1986). The toxin is well known to affect sensory neurotransmission and has analgesic properties in animals and humans, as has been demonstrated in many morphological, functional and clinical studies (see Bossowska and Majewski 2012; Kuo 2013; Pinto et al. 2013; Matak et al. 2014). Moreover, BTX appears to influence the urothelial release of adenosine triphosphate (ATP; Collins et al. 2013; Hanna-Mitchell et al. 2013). However, the proper storage and elimination of urine are also largely dependent upon the activity of the sympathetic/noradrenergic supply. The noradrenergic efferent pathways facilitate relaxation of the detrusor muscle via β-adrenoreceptors and excite the bladder base and urethra via α_1_-adrenoreceptors, thus achieving continence (Andersson and Arner 2004; Morrison et al. 2005). Notably, some information suggests that BTX can affect noradrenergic transmission in the lower urinary tract (Smith et al. 2003; Wojtkiewicz et al. 2008; Lepiarczyk et al. 2011).

The sympathetic noradrenergic innervation of the urinary bladder arises from various sources (for reviews, see Jobling 2011; de Groat and Wickens 2013) including the sympathetic chain ganglia (SChG), inferior mesenteric ganglion (IMG), pelvic ganglia (considered as mixed autonomic ganglia consisting of both sympathetic and parasympathetic cells; Keast 1992) and intramural ganglia of the urinary bladder (which contain some noradrenergic neurons; Downie et al. 1984). In females, however, SChG are considered to provide a great (or maybe the greatest) contribution to the bladder noradrenergic nerve supply and most of this innervation supplies blood vessels (Chien et al. 1991; Houdeau et al. 1998). Vera and Nadelhaft (1992) have revealed that, in the female rat, the largest part of the sympathetic innervation of the bladder dome and base arises from the SChG (77 % and 89 %, respectively), and not from the IMG.

The thorough analysis of the literature suggests that the administration of BTX into the urinary bladder wall can evoke a plastic response of bladder-projecting noradrenergic neurons, as reflected by changes in their chemical coding (Wojtkiewicz et al. 2008; Lepiarczyk et al. 2011). Studies dealing with the problem of the plasticity of particular neuronal populations have often involved combined retrograde tracing and immunohistochemistry (Kaleczyc et al. 1995; Bossowska and Majewski 2012; Pidsudko 2014). However, the information concerning the plastic response of sympathetic neurons supplying the mammalian urinary bladder to the BTX treatment gained with these methods is extremely limited. Only one congress abstract (Wojtkiewicz et al. 2008) is available reporting changes in the chemical coding of IMG urinary bladder projecting neurons (UBPN) after intravesical toxin administration in the domestic female pig and no such investigations regarding the population of UBPN in SChG have been performed so far.

Therefore the present study has been designed to investigate the chemical coding of neurons in the SChG projecting to the urinary bladder in normal (intact) pigs and in BTX-treated pigs by using combined retrograde tracing and double-labelling immunohistochemistry. As BTX is nowadays applied in the therapies of a range of neurogenic urinary bladder disorders in humans, and as the prevalence of these syndromes is clearly higher in women compared with men (Terai et al. 2004), we have examined female animals in the present research. We have decided to employ domestic pigs in our experiments, since they are one of the major animal species used in biomedical research and because they share with humans similar anatomical and physiological characteristics involving the urinary, cardiovascular, integumentary and digestive systems (e.g., Dalmose et al. 2000; Kuzmuk and Schook 2011; Swindle et al. 2012).

## Materials and methods

### Laboratory animals

The study was performed on 10 juvenile (8–12 weeks old, 15–20 kg body weight, b.w.) female pigs of the Large White Polish race. The animals were kept under standard laboratory conditions. They were fed standard fodder (Grower Plus, Wipasz, Wadąg, Poland) and had free access to water.

Before any surgical procedure were performed, all the pigs were pretreated with atropine (Polfa, Poland, 0.04 mg/kg b.w., s.c.) and propionylpromasine (Stresnil, Janssen Pharmaceutica, Belgium, 0.5 mg/kg b.w., i.m.), and after 30 min, the main anaesthetic drug, sodium pentobarbital (Tiopental, 0.5 g per animal), was given intravenously in a slow fractionated infusion. The depth of anaesthesia was monitored by testing the corneal reflex.

The animals were housed and treated in accordance with the rules of the local Ethics Commission (affiliated to the National Ethics Commission for Animal Experimentation, Polish Ministry of Science and Higher Education).

### Surgical procedures

In all the pigs, a mid-line laparotomy was performed and the urinary bladder was gently exposed to administer a total volume of 40 μl of a 5 % aqueous solution of the fluorescent retrograde tracer Fast Blue (FB; Dr K. Illing, Gross Umstadt, Germany) into the wall of its right side in multiple injections. To avoid any leakage, the needle was left in situ for about 1 min. The wall of the injected organ was then rinsed with physiological saline and gently wiped with gauze.

Three weeks later (the optimal time for the retrograde tracer to be transported to the SChG and to label UBPN; Bossowska et al. 2009), the pigs were divided into two groups. One group of 5 pigs served as controls and were injected (multiple injections) into the wall of the right side of the urinary bladder with 40 μl aqueous saline solution by using a cystoscope. Another group of 5 pigs was injected (multiple injections) into the wall of the right side of the urinary bladder with BTX type A (Botox, 100 IU, 40 μl) by using a cystoscope in order to mimic the route of its administration used in humans.

One week after the administration of the aqueous saline solution or BTX, all the pigs were deeply anaesthetized with sodium pentobarbital and transcardially perfused with 4 % buffered paraformaldehyde (pH 7.4). Three pigs studied had 14 ribs, five animals had 15 ribs and two animals had 16 ribs and, thus, they had 14, 15 or 16 thoracic (Th) SChG, respectively. All Th, lumbar (L) and sacral (S) SChG were collected (i.e. 14–16 Th, 6 L and 4 S), post-fixed in the same fixative (10 min at room temperature), washed several times in 0.1 M phosphate buffer and stored in 18 % buffered sucrose at 4 °C until being used for sectioning.

### Sectioning of ganglia and estimation of total number of SChG-UBPN

The SChG were cut with a HM525 Zeiss cryostat into 10-μm-thick serial sections. To calculate the number of FB-positive (FB+) perikarya, they were counted in every fourth section to avoid double-counting of the same neuron (most neurons were approximately 40 μm in diameter). Only neurons with a clearly visible nucleus were considered.

The total numbers of nerve cells counted in the SChG and the relative frequencies of perikarya in the ganglia from either side of each segmental level are presented as means ± standard deviation (SD).

### Immunohistochemical procedure

Immunohistochemistry involved double-staining that was performed according to a method described earlier (Bossowska and Majewski 2012) and was applied to cryostat sections from both the ipsi- and contralateral L3 and L5 ganglia and from the S2 ganglia, because these ganglia were found to contain the highest numbers of FB+ neurons. The sections were selected from three different representative regions of the ganglia (upper one-third, middle and lower one-third).

Immunohistochemical characteristics of FB+ neurons were investigated by using primary antibodies against dopamine β-hydroxylase (DβH; marker of noradrenergic neurons), neuropeptide Y (NPY), vasoactive intestinal polypeptide (VIP), somatostatin (SOM), galanin (GAL), Leu^5^-enkephalin (L-ENK) and neuronal nitric oxide synthase (nNOS; for details concerning all the primary and secondary antibodies used, see Table 1). DβH antiserum was applied in a mixture with antisera against NPY, VIP, SOM, GAL, L-ENK or nNOS. The presence of the above-mentioned active substances (NPY, VIP, SOM, GAL, L-ENK) or their markers (DβH, nNOS, namely enzymes of the catecholamine or nitric oxide biosynthesis pathway, respectively) was previously revealed in the SChG neurons of pig (Skobowiat et al. 2011; Ragionieri et al. 2013; Pidsudko 2014).

**Table 1:** List of primary antisera and secondary reagents used (*DβH* dopamine β-hydroxylase, *NPY* neuropeptide Y, *VIP* vasoactive intestinal polypeptide, *SOM* somatostatin, *GAL* galanin, *L*-*ENK* Leu^5^-enkephalin, *nNOS* neuronal nitric oxide synthase, *FITC* fluorescein isothiocyanate)

Antigen	Code	Dilution	Host	Supplier
Primary antibodies				
DβH	MAB 308	1:1000	Mouse	Millipore, USA
NPY	NA 1233	1:8000	Rabbit	Enzo Life Sciences, USA
VIP	VA 1285	1:4000	Rabbit	Enzo Life Sciences, USA
SOM	11,180	1:4000	Rabbit	ICN-Cappel, USA
GAL	AB 5909	1:4000	Rabbit	Millipore, USA
L-ENK	EA 1149	1:9000	Rabbit	Enzo Life Sciences, USA
nNOS	AB 5380	1:16,000	Rabbit	Millipore, USA
Secondary reagents				
Biotinylated anti-rabbit immunoglobulins	E 0432	1:1000	Goat	Dako, Germany
CY3-conjugated strepatvidin	711-165-152	1:13,000	–	Jackson, USA
FITC-conjugated anti-mouse IgG	715-096-151	1:700	Donkey	Jackson, USA

The application of antisera raised in various species allowed investigation of the coexistence of DβH with other substances. Retrogradely labelled and immunohistochemically investigated perikarya were evaluated under an Olympus BX61 microscope equipped with an epifluorescence filter and an appropriate filter set for CY3 and fluorescein isothiocyanate (FITC).

To determine percentages of the particular neuronal subpopulations, at least 200 FB+ neuronal profiles investigated with one combination of the primary antisera for the coexpression of the biologically active substances were counted in each ganglion studied (bilateral L3, L5 and S2); thus, at least 600 neurons were analysed for one coexistence pattern of the neurotransmitters in every animal. In both the control and BTX-treated animals, the percentages of the retrogradely labelled neurons immunopositive to particular biologically active substances or their markers investigated were pooled and presented as means ± SD.

Micrographs were taken by using an Olympus XM10 digital camera. The microscope was equipped with cellSens Dimension Image Processing software. Morphometric data relative to each neuronal class were compared within each animal and among animals and were analysed by the Student *t*-test by using GraphPad PRISM 4.0 software and by Pearson’s Chi-squared test with Yates’ continuity correction. Differences in both tests were considered to be significant at *P* < 0.05.

### Control of specificity of tracer staining and immunohistochemical procedures

Thorough macroscopic examinations of the sites of FB injections and the tissues adjacent to the urinary bladder were performed before the collection of information from the ganglia. The sites of the injections were easily identified by the yellow-labelled deposition left by the tracer within the bladder wall. Moreover, the injection sites were also observed under UV lamp rays in the dark room. The tissues adjacent to the bladder were not found to be contaminated with the tracer. To verify that the tracer had not migrated into the urethra, we analysed, in cryostat sections and by means of the haematoxylin and eosin staining technique, the junction between the urinary bladder trigone and cranial portion of the urethra. In all the animals studied, no contamination of the tracer was found within the urethra. All these procedures excluded any leakage of the tracer and validated the specificity of the tracing protocol.

**Fig. 1:**
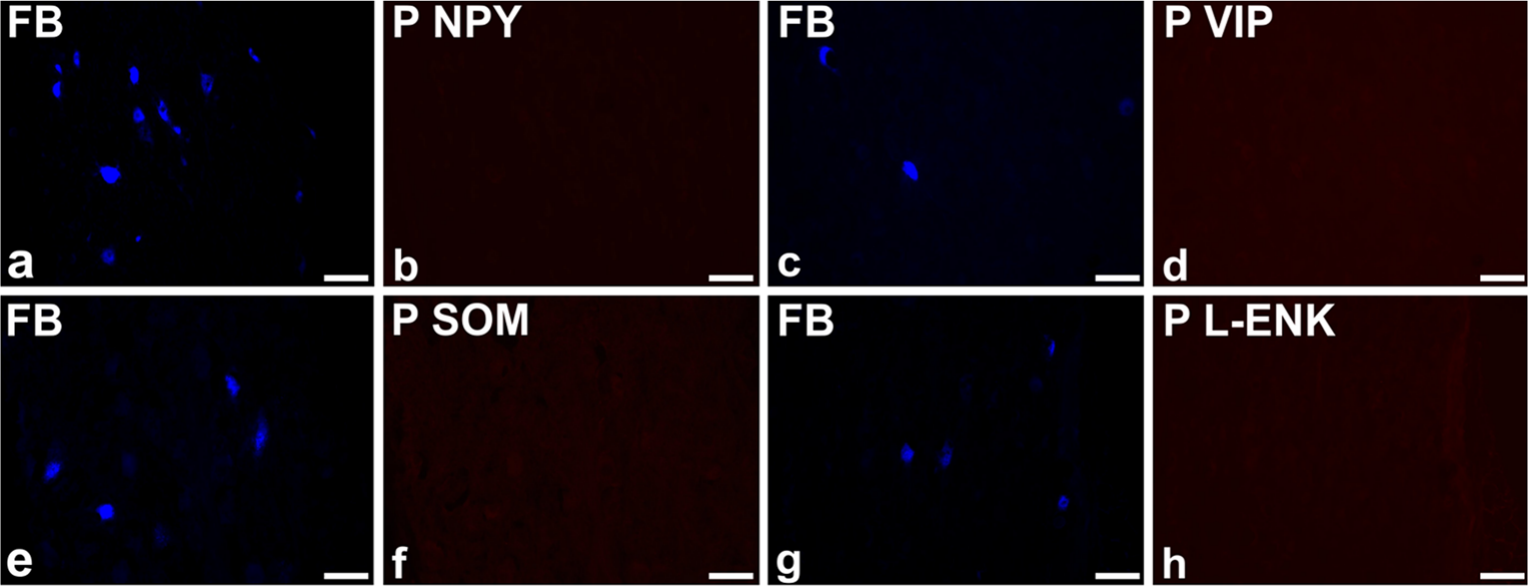
Representative images of sympathetic chain ganglia urinary bladder-projecting neurons (SChG-UBPN) in control pigs. Micrographs were taken separately from the *blue* fluorescent channel (**a**, **c**, **e**, **g**) and corresponding images taken separately from the *red* fluorescent channel demonstrating the results of the preabsorption procedure for neuropeptide Y (*P NPY*, **b**), vasoactive intestinal polypeptide (*P VIP*, **d**), somatostatin (*P SOM*, **f**) or Leu^5^-enkephalin (*P L-ENK*, **h**). The preabsorption of the specific antiserum with an appropriate antigen completely eliminated positive staining (**b**, **d**, **f**, **h**). *Bar* 100 μm (**a–d**, **g**, **h**), 50 μm (**e**, **f**)

Standard controls, i.e. preabsorption for the neuropeptide antisera (20 μg appropriate antigen per 1 ml corresponding antibody at working dilution; all antigens purchased from Peninsula, Sigma or Dianova, Fig. 1) or omission and replacement of the respective primary antiserum with the corresponding non-immune sera, completely abolished immunofluorescence and eliminated specific staining.

## Results

### Distribution of FB+ neurons in control and BTX-treated pigs

**Fig. 2:**
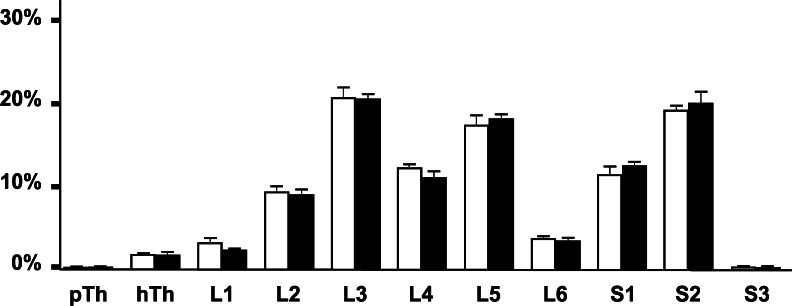
Bar diagram showing relative frequencies (%) of urinary bladder-projecting neurons in sympathetic chain ganglia (SChG) of the control pigs (*n* = 5, *open bars*) and the BTX-treated animals (*n* = 5, *solid bars*). Each *bar* represents mean ± SD of pooled data from one pair of SChG (*pTh* penultimate thoracic ganglion, *hTh* hindmost thoracic ganglion, *L* lumbar ganglion, *S* sacral ganglion)

After injection of FB into the wall of the right side of the urinary bladder, FB+ neurons were found distributed in the bilateral Th, L and S SChG, from the last two Th ganglia to the S3 ganglia in all the pigs. Distinct left-right differences were observed as the vast majority of the neurons in both control and BTX-treated animals were found in the right and thus ipsilateral ganglia. In control pigs, 1364 ± 154.6 FB+ neurons (16.5 ± 2.9 %) were found in the left SChG and 7011 ± 935.1 neurons (83.5 ± 2.9 %) were found in the right SChG. In the BTX-treated animals, 1199 ± 97.6 FB+ neurons (14.5 ± 2.1 %) were found in the left SChG and 7133 ± 652.9 FB+ neurons (85.5 ± 2.1 %) were found in the right SChG. Moreover, in both the control and experimental pigs, the right L3, L5 and S2 ganglia appeared to contain distinctly more FB+ neurons than each of the other ganglia. The relative frequency of FB+ neurons in the particular SChG of the control and BTX-treated animals is shown in Fig. 2.

The labelled neurons were isolated or sometimes clustered in small groups of 2–3 cells scattered throughout the individual ganglia. In the ganglia containing the highest number of FB+ neurons, the retrogradely labelled cells were distributed evenly. In the ganglia containing smaller numbers of FB+ perikarya, the neurons were almost exclusively distributed close to and along the ganglionic border.

### Immunohistochemical characteristics of FB+ neurons in control pigs

**Fig. 3:**
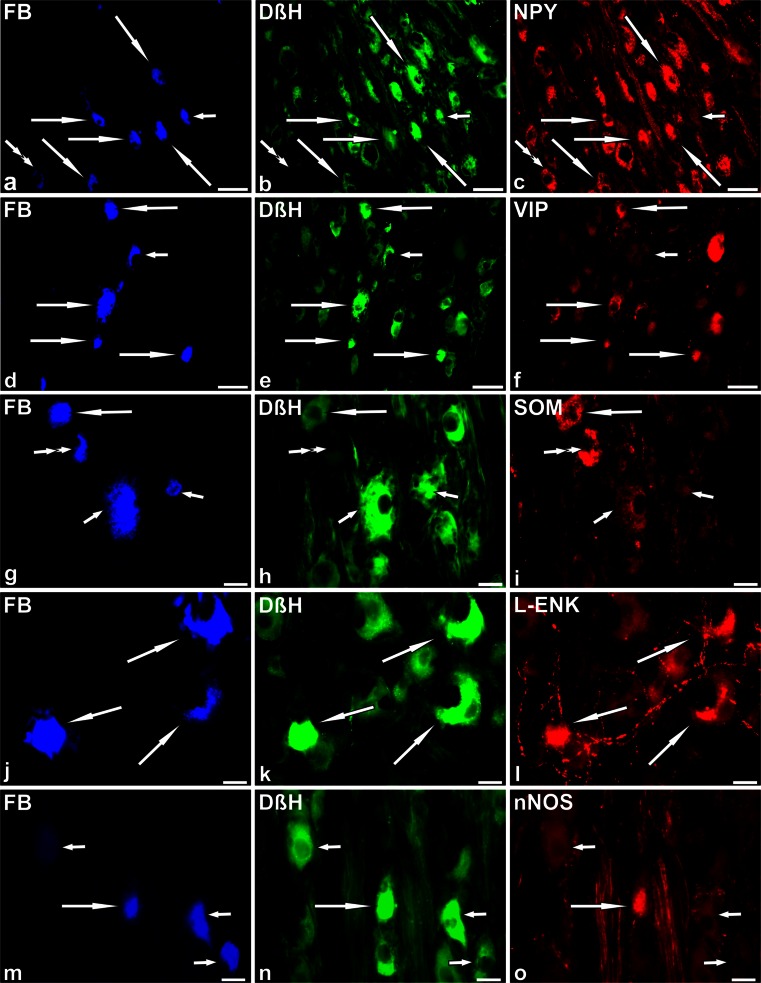
Representative images of SChG-UBPN in control pigs. All images were taken separately from *blue* (**a**, **d**, **g**, **j**, **m**), *green* (**b**, **e**, **h**, **k**, **n**) and *red* (**c**, **f**, **i**, **l**, **o**) fluorescent channels. **a–c** Seven fast blue-positive (FB+) neurons (**a**, *blue*, 5 *long arrows*, 1 *short arrow*, 1 *double arrow*), which were simultaneously DβH+ (**b**, *green*, 5 *long arrows*, 1 *short arrow*) or DβH- (1*double arrow*) and NPY+ (**c**, *red*, 5 *long arrows*, 1 *double arrow*) or NPY- (1 *short arrow*). **d–f** Five FB+ neurons (**d**, *blue*, 4 *long arrows*, 1 *short arrow*), which were simultaneously DβH+ (**e**, *green*, 4 *long arrows*, 1 *short arrow*) and VIP+ (**f**, *red*, 4 *long arrows*) or VIP- (1 *short arrow*). **g–i** Four FB+ neurons (**g**, *blue*, 1 *long arrow*, 2 *short arrows*, 1 *double arrow*), which were simultaneously DβH+ (**h**, *green*, 1 *long arrow*, 2 *short arrows*) or DβH- (1 *double arrow*) and SOM+ ( **i**, *red*, 1 *long arrow*, 1 *double arrow*) or SOM- (2 *short arrows*). **j–l** Three FB+ neurons (**j**, *blue*, 3 *long arrows*), which were simultaneously DβH+ (**k**, *green*, 3 *long arrows*) and L-ENK+ (**l**, *red*, 3 *long arrows*). **m–o** Four FB+ neurons (**m**, *blue*, 1 *long arrow*, 3 *short arrows*), which were simultaneously DβH+ (**n**, *green*, 1 *long arrow*, 3 *short arrows*) and nNOS+ (**o**, *red*, 1 *long arrow*) or nNOS- (3 *short arrows*). *Bars* 50 μm (**a–f**), 20 μm (**g–o**)

Double-labelling immunohistochemistry revealed two main populations of FB+ neurons. The vast majority (91 ± 2.3 %) of the retrogradely labelled nerve cells were DβH-positive (DβH+; Fig. 3b, e, h, k, n). The remaining FB+ neurons (9 ± 2.3 %) were DβH-negative (DβH-; Fig. 3b, h).

Among the FB+/DβH+ neurons, many (74.5 ± 11.9 %) perikarya also stained for NPY (Fig. 3a–c). Some of the FB+/DβH+ neurons were immunopositive to VIP (22.3 ± 8.8 %; Fig. 3d–f) or SOM (17.4 ± 3.7 %; Fig. 3g–i). A small number (5.4 ± 4.4 %) of FB+/DβH+ nerve cells expressed immunoreactivity to GAL. Single FB+/DβH+ neuronal somata were L-ENK-immunopositive (1.1 ± 0.8 %; Fig. 3j–l) or nNOS-immunopositive (0.8 ± 0.6 %; Fig. 3m–o).

Among FB+ non-adrenergic (DβH-) neurons, many perikarya stained for NPY (27.2 ± 12.7 %; Fig. 3a–c). Single FB+/DβH- neurons (0.6 ± 1.4 %) were SOM+ (Fig. 3g–i). No FB+/DβH- nerve cells contained immunoreactivity to VIP, GAL, L-ENK or nNOS.

### Immunohistochemical characteristics of FB+ neurons in BTX-treated animals

**Fig. 4:**
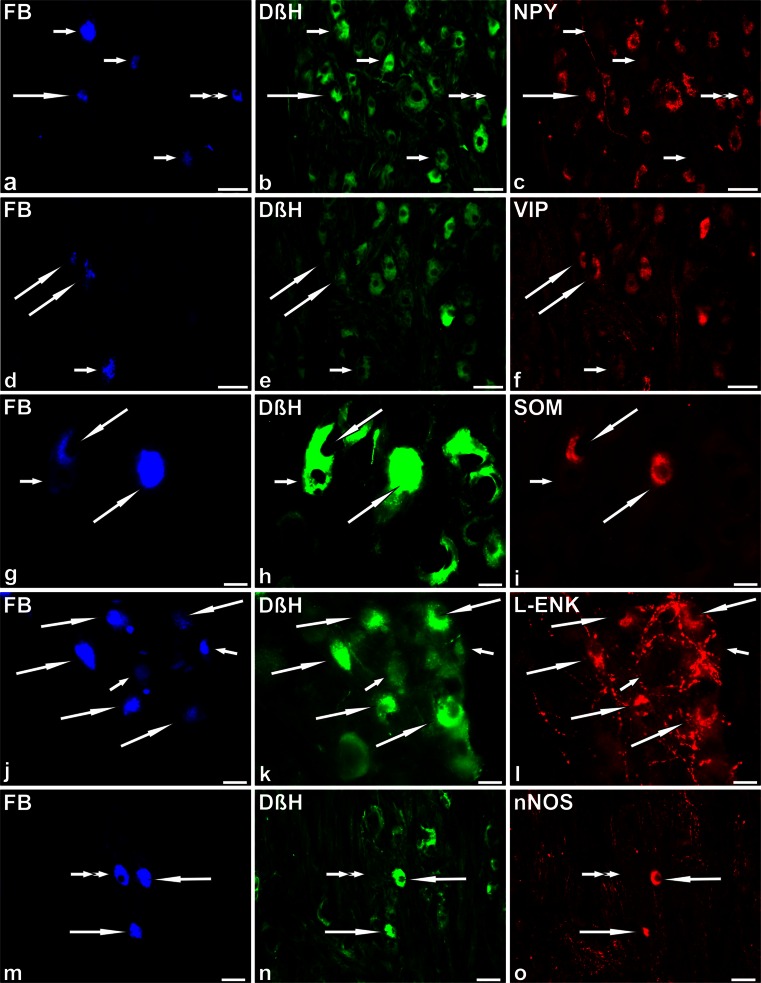
Representative images of SChG-UBPN in BTX-treated pigs. All images were taken separately from *blue* (**a**, **d**, **g**, **j**, **m**), *green* (**b**, **e**, **h**, **k**, **n**) and *red* (**c**, **f**, **i**, **l**, **o**) fluorescent channels. **a–c** Five FB+ neurons (**a**, *blue*, 1 *long arrow*, 3 *short arrows*, 1 *double arrow*), which were simultaneously DβH+ (**b**, *green*, 1 *long arrow*, 3 *short arrows*) or DβH- (1 *double arrow*) and NPY+ (**c**, *red*, 1 *long arrow*, 1 *double arrow*) or NPY- (3 *short arrows*). **d–f** Three FB+ neurons (**d**, *blue*, 2 *long arrows*, 1 *short arrow*), which were simultaneously DβH+ (**e**, *green*, 2 *long arrows*, 1 *short arrow*) and VIP+ (**f**, *red*, 2 *long arrows*) or VIP- (1 *short arrow*). **g–i** Three FB+ neurons (**g**, *blue*, 2 *long arrows*, 1 *short arrow*), which were simultaneously DβH+ (**h**, *green*, 2 *long arrows*, 1 *short arrow*) and SOM+ (**i**, *red*, 2 *long arrows*) or SOM- (1 *short arrow*). **j–l** Seven FB+ neurons (**j**, *blue*, 5 *long arrows*, 2 *short arrows*), which were simultaneously DβH+ (**k**, *green*, 5 *long arrows*, 2 *short arrows*) and L-ENK+ (**l**, *red*, 5 *long arrows*) or L-ENK- (2 *short arrows*). **m–o** Three FB+ neurons (**m**, *blue*; 2 *long arrows*, 1 *double arrow*), which were simultaneously DβH+ (**n**, *green*, 2 *long arrows*) or DβH- (1 *double arrow*) and nNOS+ (**o**, *red*, 2 *long arrows)* or nNOS- (1 *double arrow*). *Bars* 50 μm (**a–f**, **m–o**), 20 μm (**g–l**)

Double-labelling immunohistochemistry revealed that, similar to the control group, the vast majority of FB+ UBPN (89.8 ± 2.5 % in BTX-treated animals vs 91 ± 2.3 % in the control group) were DβH+ (Fig. 4b, e, h, k, n). The remaining nerve cells (Fig. 4b, n) were also DβH- (10.2 ± 2.5 % in BTX-treated animals vs 9 ± 2.3 % in the control group).

**Fig. 5:**
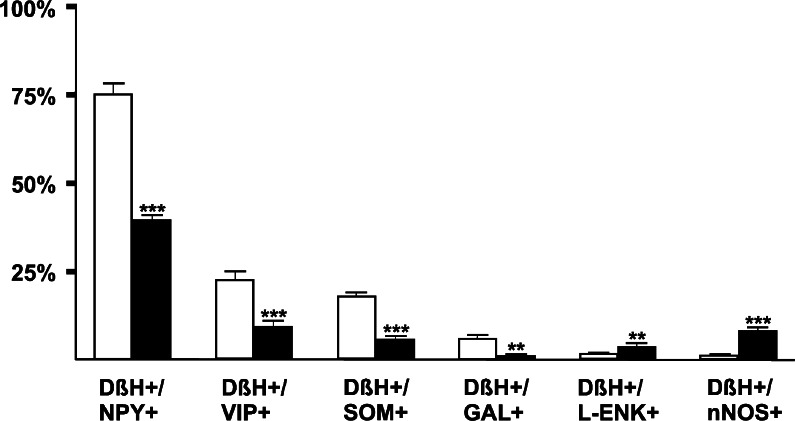
Bar diagram showing relative frequencies (%) of subpopulations of noradrenergic (DβH+) retrogradely labelled (FB+) bladder-projecting neurons in sympathetic chain ganglia (SChG) of control pigs (*n* = 5, *open bars*) and BTX-treated animals (*n* = 5, *solid bars*). Each *bar* represents mean ± SD of pooled data from bilateral L3, L5 and S2 SChG (*DβH* dopamine β-hydroxylase, *NPY* neuropeptide Y, *VIP* vasoactive intestinal polypeptide, *SOM* somatostatin, *GAL* galanin, *L-ENK* Leu^5^-enkephalin, *nNOS* neuronal nitric oxide synthase). Statistically significant differences: **P ≤ 0.01, ***P ≤ 0.005

However, in BTX-treated pigs, the percentages of FB+ noradrenergic neuronal subpopulations distinctly varied from those determined in the control animals (Fig. 5). A lower number of FB+/DβH+/NPY+ neurons (39.5 ± 4.5 % vs 74.5 ± 11.9 %) were observed in BTX-treated pigs (Fig. 4a–c). Furthermore, in the toxin-injected animals a decrease in the number of FB+/DβH+ neurons immunopositive to VIP (8.9 ± 5.3 % vs 22.3 % ± 8.8 %; Fig. 4d–f), SOM (5.8 ± 2.3 % vs 17.4 ± 3.7 %; Fig. 4g–i) or GAL (0.9 ± 1.2 % vs 5.4 ± 4.4 %) was observed. On the other hand, in BTX-injected pigs, an increase in the number of the noradrenergic UBPN immunoreactive to L-ENK (3.7 ± 2.9 % vs 1.1 ± 0.8 %; Fig. 4j–l) or nNOS (7.7 ± 3.5 % vs 0.8 ± 0.6 %; Fig. 4m–o) was observed.

In FB+/DβH- neurons, BTX treatment was followed by a visible decrease in NPY+ (Fig. 4a-c) or SOM+ perikarya (8.4 ± 3.3 % vs 27.2 ± 12.7 % and 0 % vs 0.6 ± 1.4 %, respectively). As in the control pigs, no FB+/DβH- neurons containing immunoreactivity to VIP, GAL, L-ENK or nNOS were encountered.

## Discussion

The present results indicate that the SChG should be considered an important source of the autonomic innervation of the urinary bladder in the female pig. This assumption corresponds well with observations of other authors (Chien et al. 1991; Vera and Nadelhaft 1992; Houdeau et al. 1998) dealing with the contribution of SChG to the innervation of various internal organs including the bladder in the female rat. We should emphasize that no morphological studies on the distribution and chemical coding of SChG-UBPN in intact female pigs have been performed so far. However, two such investigations have been carried out in male animals (Ragionieri et al. 2013; Pidsudko 2014). The distribution of SChG-UBPN in male and female pigs is generally comparable. Small differences observed between males and females might result from the different allocation of injection sites of FB chosen in the experiments or might represent sex-dependent variations.

Pidsudko (2014) has found 1083 ± 291.5, 1581 ± 71.44 and 250 ± 14.29 FB+ neurons in the SChG after the administration of tracer to the bladder trigone, cervix or apex, respectively, which together account for about 32 % of sympathetic nerve cells involved in the innervation of the organ. These numbers of neurons are smaller in comparison with those determined in the present study (together 8226 ± 1069 FB+ neurons in control pigs). As mentioned, this divergence is most probably a consequence of a difference in the allocation of the FB injection sites employed in the experiments. Notably, Wakabayashi et al. (1994) have found that adrenergic nerve fibres richly supply not only the bladder base, but also the bladder body (which was not injected with a tracer by Pidsudko 2014). The other possible explanation might be related to sex-dependent variations.

In this study, no significant differences in the number and distribution of UBPN have been observed between the intact and BTX-treated pigs. This finding corresponds well with the observation of Apostolidis et al. (2005) who have demonstrated that the application of BTX into the human bladder detrusor muscle has no influence on the number of suburothelial nerve fibres visualized by immunohistochemistry with an antibody to pan-neuronal marker protein gene product 9.5 (PGP 9.5) in bladder biopsies taken after toxin administration. We can therefore conclude that, at least in both humans and pigs, the mechanism of BTX action on the bladder is not associated with neuronal death.

The chemical coding of porcine FB+ SChG-UBPN determined in this study is generally comparable with that observed by other authors in the male pig (Ragionieri et al. 2013; Pidsudko 2014). The vast majority of the retrogradely labelled nerve cells in both females (91 ± 2.3 %) and males (90 %; Pidsudko 2014) are DβH+ suggesting their noradrenergic nature. This observation is not surprising because the SChG consist of mostly noradrenergic neurons providing a prominent contribution to the noradrenergic nerve supply to various internal organs (Kummer 1987; Heym et al. 1990; Skobowiat et al. 2011). Many of the noradrenergic neurons observed in the present study exhibit immunoreactivity to NPY and some of them are immunostained for VIP, SOM, GAL, nNOS or L-ENK. In general, similar biologically active substances have been determined by Ragionieri et al. (2013) and Pidsudko (2014) in porcine male SChG-UBPN and by other authors (Hill and Elde 1989; Łakomy et al. 1994; Majewski 1999; Skobowiat et al. 2011) in porcine SChG noradrenergic neurons.

The present findings suggest that BTX is a factor evoking strong adaptation changes in SChG-UBPN. We should emphasize that no morphological investigations dealing with the influence of BTX on the chemical coding of SChG neurons innervating the mammalian urinary bladder have been performed previously. However, Wojtkiewicz et al. (2008) have investigated BTX-induced changes in the chemical features of IMG neurons supplying the porcine urinary bladder.

In the present study, the percentages of DβH+ SChG-UBPN have been found to be similar in both the control and BTX-treated pigs (91 ± 2.3 % and 89.8 ± 2.5 %, respectively). We can therefore conclude that the toxin does not affect the noradrenergic profile of these nerve cells. This observation corresponds well with results of a previous study (Lepiarczyk et al. 2011) that has revealed that BTX-treatment does not decrease the number of DβH+ nerve fibres distributed in the wall of the porcine female urinary bladder. As mentioned before, the release of the sympathetic neurotransmitter noradrenaline (NA) allows continence to be achieved (Andersson and Arner 2004; Morrison et al. 2005). Thus, the unchanged relative frequency of noradrenergic neurons observed in the present study can be considered as an advantageous finding from the perspective of the use of BTX in the treatment of urinary bladder disorders involving the overactivity of the bladder detrusor and might be the reason for good clinical results.

Although the numbers of noradrenergic SChG-UBPN are similar in the intact and BTX-treated pigs, the present study has revealed that the expression of some biologically active substances in these nerve cells distinctly differ between the two groups. In general, the administration of the toxin results in a significant decrease in the number of the noradrenergic neurons expressing NPY, VIP, SOM and GAL but an increase in the number of the neurons stained for L-ENK and nNOS. Similar results have been obtained by Wojtkiewicz et al. (2008) who have observed a significant decrease in the number of NPY+ and VIP+ noradrenergic UBPN in the porcine female IMG after the application of BTX into the wall of the organ. However, in contrast to the present findings, Wojtkiewicz et al. (2008) have found an increase in the number of the neurons expressing SOM.

The changes in the expression of NPY, VIP and SOM in particular are intriguing, because immunoreactivities to these substances have been found in many noradrenergic SChG-UBPN in normal (intact) pigs and, thus, the role that they play can also be considered as proportionally significant. We should also keep in mind that all the substances mentioned are expressed in noradrenergic neurons, thus, in both the intact and BTX-treated animals, they most probably function as neuromodulators or co-transmitters in relation to the main neurotransmitter, NA. As previously mentioned, the positive effect of BTX on urinary bladder disorders in humans is thought, in general, to be associated with a withdrawal of the cholinergic input and thus with the achievement of the prevalence of the noradrenergic involvement. We are therefore tempted to assume that the significance of the changes in the expression of the various active substances in the noradrenergic neurons observed in the present study is to accomplish the enhancement of the contribution of the noradrenergic nerve supply. This hypothesis is supported by some interesting observations dealing with the influence of NPY on noradrenergic transmission in urogenital organs. Tran et al. (1994) have found that this peptide exerts a prejunctional inhibitory effect on noradrenergic transmission in the rat lower urinary tract. Moreover, NPY has been established to depress the release of NA from nerves supplying the rat vas deferens (Lundberg and Stjarne 1984; Bitran et al. 1996) and seminal vesicle (Iravani and Zar 1994) or rabbit oviduct (Chernaeva and Charakchieva 1988). The decrease in the expression of NPY in the subpopulation of noradrenergic neurons observed in the present study can therefore be assumed to result in the reduction of the inhibitory effect of this peptide on noradrenergic transmission and thus to improve continence. The significance of a decrease in the expression of VIP, SOM and GAL is probably also involved with the enhancement of NA release. The literature in the field contains information justifying this idea, however, the relevant data have not been obtained in studies performed on urinary tract tissues. VIP has been found to inhibit the release of NA from nerves supplying the rat portal vein (Bråtveit and Helle 1991). SOM has been revealed to inhibit the release of NA from rat hypothalamic neurons (Göthert 1980), rat mesenteric artery (Calhau et al. 2000) and rabbit ear artery (Maynard et al. 1991). GAL (together with NPY) has been observed to significantly inhibit the stimulation-evoked NA release in the rat hypothalamus (Tsuda et al. 1989) and medulla oblongata (Tsuda et al. 1992).

On the other hand, an increase in the expression of nNOS and L-ENK suggests that both nitric oxide (NO) and L-ENK can act as co-transmitters supporting NA in affecting bladder smooth muscle contraction. NO, a substance well recognized as also exerting smooth muscle relaxation in urogenital organs (Persson et al. 1993; Burnett 1995) can be assumed to potentiate the relaxatory effect of NA on the detrusor muscle mediated via β adrenergic receptors. Opioid peptides, in turn, have been found to evoke rapid long-lasting contractile effects on the female rat intrinsic urethral sphincter (Crayton et al. 2010). Because, as mentioned before, NA also excites the bladder base and urethra musculature via α_1_-adrenoreceptors, L-ENK can be considered to assist NA in this action.

The present study has revealed a small (9 ± 2.3 % in the control group vs 10.2 ± 2.5 % in BTX-treated animals) population of non-adrenergic (DβH-) SChG-UBPN. Many of these nerve cells immunostain for NPY and the solitary neuronal somata are SOM+. The most significant finding involving the chemical coding of these neurons is a distinctly lower number of especially NPY+ perikarya in BTX-treated pigs. We should mention that Pidsudko (2014) has revealed that small numbers of SChG-UBPN in the male pig express VAChT (a cholinergic marker) or neither VAChT nor DβH. We can therefore assume that, in the female pig, BTX affects the cholinergic (or even non-adrenergic, non-cholinergic) transmission in SChG-UBPN via the down-regulation of NPY expression.

In conclusion, the present study has revealed the existence of profound differences in the chemical coding of the SChG-UBPN between normal female pigs and pigs after intravesical BTX injections. The most important findings include a significant decrease in the number of the noradrenergic neurons expressing NPY, VIP, SOM and GAL and an increase in the number of the neurons expressing nNOS and L-ENK. We can hypothesize that, in the toxin-administered animals, these changes cause the enhancement of the noradrenergic input to the bladder tissues thereby constituting an additional (to that involving the cholinergic transmission) beneficial effect of BTX treatment. Therefore, the present study has, for the first time, provided evidence that the therapeutic effects of BTX on the mammalian urinary bladder is partly mediated by SChG neurons.
